# Carabrone inhibits *Gaeumannomyces tritici* growth by targeting mitochondrial complex I and destabilizing NAD⁺/NADH homeostasis

**DOI:** 10.1371/journal.ppat.1013567

**Published:** 2025-10-03

**Authors:** Xingyu Ren, Jing Bai, Yingying Han, Jiaying Xu, Yingchen Liu, Zhiqing Ma, Yong Wang, Juntao Feng

**Affiliations:** 1 College of plant protection, Northwest A&F University, Yangling, Shaanxi China; 2 Engineering and Research Center of Biological Pesticide of Shaanxi Province, Northwest A&F University, Yangling, Shaanxi China; USDA-ARS Plains Area, UNITED STATES OF AMERICA

## Abstract

The excessive and irrational use of commercial fungicides has led to escalating drug resistance in phytopathogens, necessitating the discovery of novel antifungal targets and strategies. Plant secondary metabolites, serving as natural chemical defenses against pathogen invasion, offer promising scaffolds and potential targets for developing innovative crop protection approaches. This study elucidates the antifungal mechanism of the natural sesquiterpene lactone carabrone against *Gaeumannomyces tritici* through integrated multi-omics analyses. Time-series transcriptomic profiling revealed that carabrone significantly suppresses the oxidative phosphorylation (OXPHOS) pathway and disrupts nicotinate/nicotinamide metabolism, resulting in a reduced NAD⁺/NADH (NAD^+^, Oxidized nicotinamide adenine dinucleotide; NADH, Reduced nicotinamide adenine dinucleotide) ratio. Orthogonal elevation of NAD⁺ levels through exogenous supplementation diminished fungal susceptibility to carabrone, establishing a direct link between NAD⁺/NADH homeostasis and its antifungal activity. Activity-based protein profiling (ABPP), gene silencing screens, and physiological-biochemical validations collectively demonstrated that carabrone specifically inhibits the electron transport chain (ETC) rather than ATP synthase to regulate NAD⁺/NADH balance. Further evidence from pyruvate supplementation, expression of the yeast non-proton-pumping NADH dehydrogenase *Scndi1*, and enzymatic assays confirmed that carabrone directly targets mitochondrial respiratory chain complex I, thereby destabilizing NAD⁺/NADH homeostasis and suppressing *G. tritici* growth. This work first establishes complex I as the direct antifungal target of carabrone, revealing its lethal mechanism involving complex I inhibition-mediated blockade of NADH oxidation, followed by oxidative stress induction and energy metabolism collapse. Additionally, we demonstrate that *Scndi1* serves as a critical tool for screening and validating complex I-targeted fungicides. These findings provide both a lead scaffold for developing novel complex I inhibitors and a systematic framework for antifungal agent validation, offering theoretical support to combat emerging fungal resistance challenges.

## Author summary

The escalating fungicide resistance crisis necessitates the discovery of novel targets for developing next-generation antifungal agents. Mitochondrial respiratory chain, as a central hub for energy metabolism, represents a critical target in fungicide development. However, commercial fungicides predominantly target complexes II/III, with scarce agents acting on complex I—the entry point of mitochondrial respiratory chain. Complex I’s structural complexity and functional importance offer unique advantages as an antifungal target, yet its functional characterization in phytopathogenic fungi remains poorly understood, hindering targeted drug development. Herein, we identified carabrone, a natural sesquiterpene lactone from medicinal plants, exhibiting novel structure and potent antifungal activity. Mechanistic studies revealed that carabrone specifically targets complex I in *Gaeumannomyces tritici*, disrupting NAD⁺/NADH homeostasis and triggering oxidative stress coupled with energy metabolism collapse, ultimately suppressing pathogen growth. Our findings not only provide a structural scaffold for complex I-targeted fungicide development but also establish *Scndi1* as a theoretical basis for screening and validating complex I inhibitors in plant pathogens.

## Introduction

Plant pathogenic fungi are critical biological agents threatening global food security and ecological balance, causing annual crop yield losses of up to 20–30% [1,2]. However, the increasing complexity of global climate change is exacerbating the risks of plant diseases to agricultural systems. Currently, chemical control remains the primary strategy for disease management. Nevertheless, the prolonged overreliance on conventional fungicides has led to escalating challenges, including widespread fungal resistance, environmental persistence, and off-target toxicity [3,4]. Mitochondrial respiratory chain-targeting fungicides, such as quinone outside/inside inhibitors (QoIs/ QiIs) and succinate dehydrogenase inhibitors (SDHIs), have demonstrated high efficacy in early applications [5–7]. However, their reliance on single target sites has resulted in the rapid emergence of resistant fungal strains [8]. These limitations underscore the urgent need to identify novel molecular targets and develop eco-friendly fungicides with reduced resistance risks to address evolving plant protection challenges.

Plant secondary metabolites serve as chemical defense arsenals evolved by plants to combat pathogen invasion and constitute a natural molecular reservoir for developing novel eco-friendly pesticides [9]. Sesquiterpene lactones (SLs), a prominent class of these metabolites, have emerged as a research focus in antifungal agent discovery due to their structural diversity and multi-target antimicrobial properties [10–12]. Carabrone, an SL isolated from *Carpesium macrocephalum* (Asteraceae), exhibits exceptional inhibitory activity against critical phytopathogenic fungi, including *G. tritici* (causal agent of wheat take-all) and *Botrytis cinerea*, primarily by targeting mitochondrion, inducing mitochondrial dysfunction and oxidative stress-triggered apoptosis [13–18]; Previous studies have also shown that carabrone exerts differential effects on mitochondrial respiratory chain enzyme activities both in vitro and in vivo, particularly the inhibition of complex I, III, I+III, and II+III activities [17,18]. Moreover, carabrone exhibits activities such as anticancer effects and amelioration of steatohepatitis [19,20]. However, similar to most SLs, the precise molecular target(s) of carabrone remain elusive, which poses a major obstacle to rational structure-based optimization and practical application of SL-derived fungicides.

The mitochondrial respiratory chain, a central hub of energy metabolism in eukaryotes, has emerged as a critical target for novel fungicide discovery due to its functional conservation and multi-target potential [21]. While inhibitors targeting complex II (succinate-ubiquinone reductase) and complex III (ubiquinol-cytochrome *c* reductase) have been successfully commercialized, the escalating resistance crisis driven by target-site mutations underscores the limitations of these conventional targets [5,6]. In addition, the alternative oxidase also plays a crucial role in mediating resistance to complex III fungicides. For example, in *G. tritici*, the alternative oxidase couples with cytochrome c oxidase to maintain electron transport in the presence of complex III inhibitors [22,23]. In contrast, mitochondrial complex I (NADH-ubiquinone oxidoreductase), the entry enzyme of the electron transport chain (ETC), offers unique advantages for fungicide design owing to its structural complexity (comprising over 45 subunits) and functional versatility—catalyzing NADH oxidation coupled with proton translocation to establish the electrochemical gradient [24,25]. This seems to provide a novel approach for the control of plant pathogenic fungi such as *G. tritici* by targeting the mitochondrial respiratory chain. However, only a limited number of complex I-targeting fungicides, such as diflumetorim, tolfenpyrad, and fenazaquin, are currently available [26]. The development and validation of novel complex I inhibitors face significant challenges due to the scarce understanding of its functional architecture in plant-pathogenic fungi.

Herein, we elucidate the antifungal mechanism of carabrone by targeting NAD^+^/NADH (NAD^+^, Oxidized nicotinamide adenine dinucleotide; NADH, Reduced nicotinamide adenine dinucleotide) homeostasis disruption in *G. tritici*. Combining transcriptomics, activity-based protein profiling (ABPP), and orthogonal NAD^+^ salvage pathway validation, we identified carabrone as a novel fungicide candidate targeting mitochondrial complex I. This work provides a foundation for developing carabrone-derived fungicides and advances a strategy to screening and validate complex I-targeting antifungals.

## Result

### Carabrone significantly affects the oxidative phosphorylation pathway in *G. tritici*

**Fig 1:**
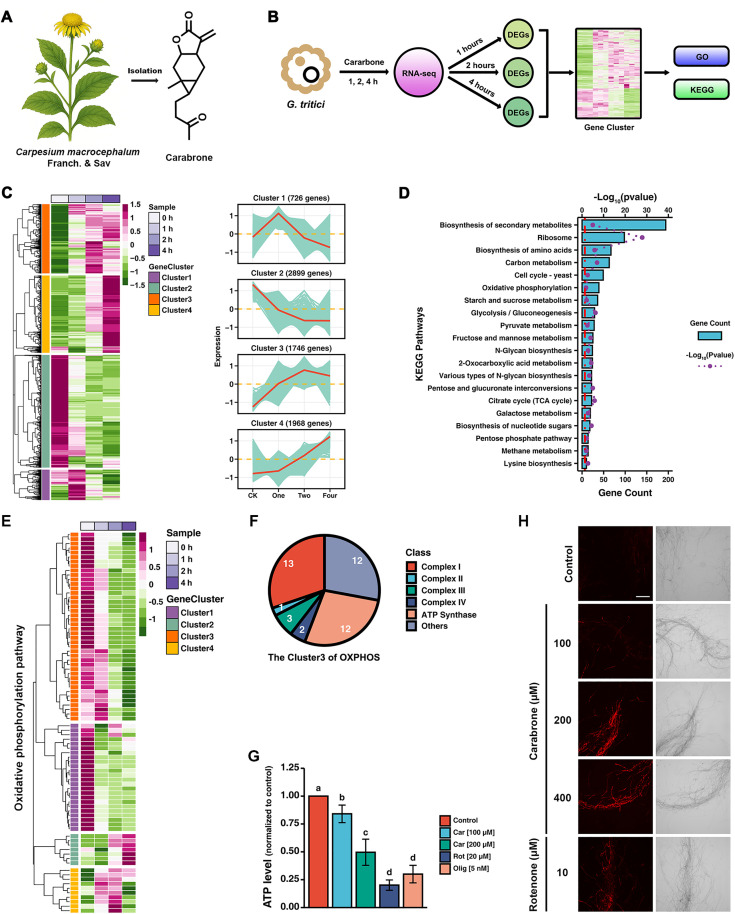
Time-series transcriptomic analysis of carabrone-treated *Gaeumannomyces tritici.* **(A)** The structure of carabrone. **(B)** Schematic illustration of time-series transcriptomic analysis. **(C)** Clustering and expression trend analysis of differentially expressed genes (DEGs). **(D)** Kyoto encyclopedia of genes and genomes (KEGG) enrichment analysis of gene cluster 2. **(E)** Heatmap of gene expression clusters in the oxidative phosphorylation (OXPHOS) pathway. **(F)** Gene assignment of cluster 3 within the OXPHOS pathway. **(G)** The ATP level of *G. tritici* treatment by carabrone (100 and 200 μM), rotenone (20 μM) and oligomycin A (5 nM). **(H)** The O_2_^.-^ level of *G. tritici* treatment by carabrone (100 and 200 μM) and rotenone (10 μM). Data are mean ± SD of *n* = 3 biologically independent experiments. Statistical significance was determined by one-way ANOVA with Tukey’s post hoc test (*P* < 0.05).

Carabrone is a sesquiterpene lactone compound, and its *α*-methylene-*γ*-butyrolactone structure is the primary functional group responsible for its antifungal activity (Fig 1A) [13,14]. It is capable of forming stable covalent bonds with cysteine residues in target proteins [27]. However, as the reaction time increases, its potential off-target effects may lead to toxicity concerns, while also adding complexity to the investigation of its specific targets [28]. Therefore, investigating the early effects of carabrone treatment on pathogenic fungi can more effectively elucidate its primary mechanism of action.

Based on this, a time-series transcriptome analysis was conducted to examine the early effects of carabrone treatment on *G. tritici* (Fig 1B). Carabrone treatment significantly affected the transcriptional levels of *G. tritici* (S1A and S1B Fig). As the treatment duration increased, both the number of differentially expressed genes (DEGs) and the magnitude of their changes gradually increased, exhibiting a pronounced time-dependent trend (Figs 1C, S1C and S1D). The gene cluster2 attracted our attention, as it exhibited a significant downregulation trend with increasing carabrone treatment time, encompassing 2,899 genes (Fig 1C). Kyoto encyclopedia of genes and genomes (KEGG) and Gene Ontology (GO) Enrichment analysis revealed significant enrichment of mitochondrial components and the oxidative phosphorylation (OXPHOS) pathway, suggesting that carabrone has a notable impact on mitochondrial function (Figs S1E and 1D). Guided by this finding, we further analyzed the expression patterns of OXPHOS-related genes among the DEGs. As expected, carabrone significantly downregulated OXPHOS-related genes, with gene cluster3 (OXPHOS pathway) showing a clear time-dependent trend (Fig 1E). Interestingly, complex I and ATP synthase components were the predominant constituents (Fig 1F). Mitochondria-related indicators were consistent with transcriptomic results. Carabrone significantly reduced ATP levels and induced superoxide anion production in *G. tritici* (Fig 1G and 1H). Collectively, these findings demonstrate that carabrone significantly affects OXPHOS at the early stage of treatment. Moreover, previous studies have also demonstrated a significant impact on OXPHOS at later stages (6–24 h) of carabrone treatment [17,18].

### Carabrone significantly impacts the NAD^+^/NADH homeostasis in *G. tritici*

**Fig 2:**
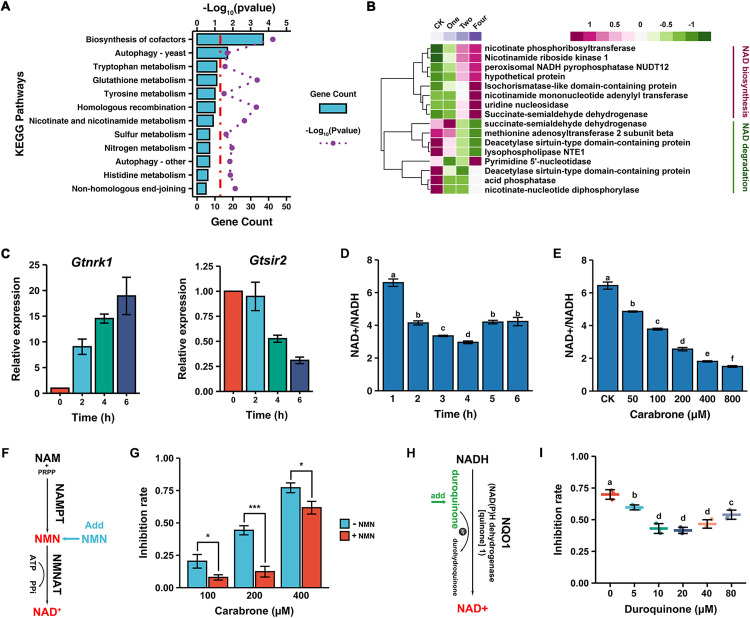
Analysis of the correlation between NAD and the antifungal activity of carabrone. **(A)** The KEGG enrichment analysis of gene cluster 4 in total DEGs. **(B)** Heatmap of DEGs in the nicotinate and nicotinamide metabolism pathway. **(C)** RT-qPCR analysis of *Gtnrk1* and *Gtsir2* in *G. tritici* after carabrone (200 μM) treatment. **(D)** NAD⁺/NADH ratio in *G. tritici* after carabrone (200 μM) treatment at different time points. **(E)** NAD⁺/NADH ratio in *G. tritici* after carabrone treatment with different concentration (treatment at 4 **h)**. **(F)** Schematic illustration of nicotinamide mononucleotide (NMN) supplementation can increase NAD^+^ synthesis by nicotinamide mononucleotide adenylyltransferase (NMNAT) activity. **(G)** Modulation of carabrone’s (200 and 400 μM) antifungal activity by NMN (400 μM) supplementation. **(H)** Schematic illustration of duroquinone can oxidize NADH to yield NAD^+^ and durohydroquinone by the activity of NAD(P)H dehydrogenase, quinone 1 (NQO1). **(I)** Modulation of carabrone’s (400 μM) antifungal activity by duroquinone supplementation. Data are mean ± SD of *n* = 3 biologically independent experiments. Statistical significance was determined by one-way ANOVA with Tukey’s post hoc test (*P* < 0.05).

DEGs in Cluster 4 exhibited a pronounced upregulation trend following carabrone treatment at different time points, which drew our attention (Fig 1C). KEGG enrichment analysis revealed that the cofactor biosynthesis pathway was significantly enriched (Fig 2A). Further investigation into all DEGs involved in the cofactor biosynthesis pathway showed that the gene cluster with an upregulation trend was significantly enriched in the vitamin biosynthetic and metabolic process (S2A and S2B Fig). Additionally, the nicotinate and nicotinamide metabolism pathway were also significantly enriched (Figs 2A and S2C). Interestingly, both of these processes and pathways are closely associated with NAD^+^. Analysis of the nicotinate/nicotinamide metabolism pathway revealed that NAD^+^ biosynthesis genes (e.g., *Gtnrk1*) showed increased expression over time with prolonged carabrone treatment, while NAD^+^ degradation genes (e.g., *Gtsir2*) exhibited decreased expression (Fig 2B and 2C). As expected, the NAD^+^/NADH ratio in *G. tritici* showed a time-dependent and concentration-dependent reduction after carabrone treatment (Fig 2D and 2E). Evidence indicates that carabrone significantly disrupts NAD⁺/NADH homeostasis in *G. tritici*.

### The antifungal activity of carabrone is closely related to NAD^+^

Nicotinamide adenine dinucleotide exists in two forms, including the oxidized (NAD^+^) and reduced (NADH) states, and plays a critical role in intermediate metabolism by serving as a cofactor in various oxidation/reduction reactions [29]. The disruption of NAD^+^/NADH homeostasis not only impacts the redox balance of cells but also directly affects cellular energy metabolism processes, such as the tricarboxylic acid (TCA) cycle and OXPHOS [30,31]. Transcriptomic analysis has demonstrated that carabrone can significantly downregulate pathways such as OXPHOS, TCA, and glycolysis (Fig 1D). Based on this, it is speculated that NAD^+^/NADH homeostasis may play a crucial role in the antibacterial activity of carabrone.

To validate this hypothesis, we tested whether altering the orthogonal NAD^+^/NADH modulating pathways affects the antifungal activity of carabrone. Nicotinamide mononucleotide (NMN) is a key precursor in the NAD^+^ salvage pathway in cell, where it is converted into NAD^+^ by NMN adenylyltransferase (NMNAT) (Fig 2F) [32–34]. As expected, exogenous addition of NMN markedly decreased the sensitivity of *G. tritici* to carabrone (Figs 2G and S2D). NAD^+^ and NADH, as cofactors for various oxidoreductases, maintain redox homeostasis through their interconversion between oxidized and reduced states. NAD(P)H dehydrogenase [quinone] 1 (NQO1) is capable of reducing duroquinone to durohydroquinone, and in the process, it consumes NADH to produce NAD^+^ (Fig 2H) [35]. As the results show (Figs 2I and S2E), the addition of different concentrations of duroquinone to the culture medium significantly reduced the sensitivity of *G. tritici* to carabrone. These results confirm that NAD^+^/NADH homeostasis is directly related to the antifungal activity of carabrone, and the imbalance in NAD^+^ levels may be a key contributing factor. This also brings our focus to NAD^+^-related proteins.

### Carabrone binds to NAD-related proteins

**Fig 3:**
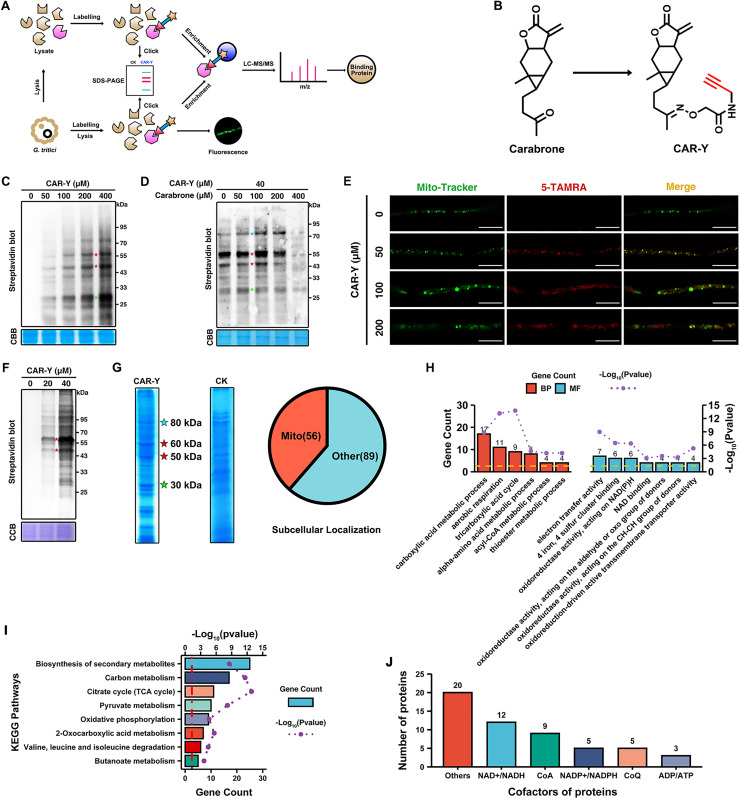
Identification of the antifungal target of carabrone by activity-based protein profiling (ABPP). **(A)** Schematic illustration of carabrone target protein identification in *G. tritici* by ABPP. **(B)** The structure of alkyne-tagged carabrone probe (CAR-Y). **(C)** In *vivo* labeling of CAR-Y in *G. tritici*, CAR-Y treatment for 4 hours. **(D)** In *vivo* competitive labeling with CAR-Y in *G. tritici*, carabrone pretreatment (4 h) followed by CAR-Y labeling in *vitro*. **(E)** Colocalization analysis of CAR-Y and mitochondria in *G. tritici*, Bar = 10 μm. **(F)** In *vitro* labeling of CAR-Y in mitochondria protein of *G. tritici*. **(G)** Enrichment and screening of CAR-Y-binding proteins in *G. tritici*. **(H)** Gene ontology (GO) enrichment analysis of potential carabrone target proteins (56 proteins) in *G. tritici*. **(I)** KEGG enrichment analysis of potential carabrone target proteins (56 proteins) in *G. tritici*. **(J)** Cofactor analysis of potential carabrone target proteins (56 proteins) in *G. tritici*. CCB: Coomassie brilliant blue. Data are mean ± SD of *n* = 3 biologically independent experiments. Statistical significance was determined by one-way ANOVA with Tukey’s post hoc test (*P* < 0.05).

To elucidate the antifungal mechanism of carabrone, we employed ABPP to identify its potential target proteins (Fig 3A). We first designed and synthesized a carabrone-based alkyne probe (CAR-Y), which exhibited no significant change in antifungal activity compared to carabrone (Figs 3B, S3A and Table 1). This indicates that CAR-Y can be effectively used for identifying the antifungal target proteins of carabrone. In *vitro* labeling experiments demonstrated that CAR-Y exhibited concentration-dependent labeling of total proteins from *G. tritici*, with significant labeling observed for proteins at approximately 30 kDa, 50 kDa, and 60 kDa (S3B Fig). Notably, the labeling of these proteins by CAR-Y could be competitively reduced by carabrone, demonstrating that the proteins labeled by CAR-Y are consistent with those bound by carabrone (S3C Fig). To enhance the reliability of protein labeling by CAR-Y, live mycelium was directly treated with CAR-Y for in *vivo* labeling. Time-dependent labeling experiments showed that treatment with 200 μM CAR-Y for 4 hours yielded satisfactory labeling efficiency (S3D Fig). Based on this result, subsequent in *vivo* labeling experiments were conducted using a 4-hour treatment period. Encouragingly, the in *vivo* labeling results were consistent with those obtained from in vitro labeling. CAR-Y exhibited concentration-dependent labeling of total proteins from *G. tritici*, with significant labeling observed not only at approximately 30 kDa, 50 kDa, and 60 kDa but also at 80 kDa (Fig 3C). Furthermore, the labeling of these proteins could be competitively inhibited by carabrone, confirming that the proteins labeled by CAR-Y are consistent with those bound by carabrone (Fig 3D).

**Table 1:** The inhibitory activities of carabrone and CAR-Y against *G. tritici.*

Compounds	Virulence equation	EC_50_ (µg/mL)	χ^2^	R^2^	95% Confidence limits
Carabrone	y = 1.794x – 3.065	51.14	5.7	0.994	43.113-62.507
CAR-Y	y = 0.965x – 2.160	62.01	3.1	0.965	48.865-81.214

Thanks to the expandability of the CAR-Y conjugation tag, 5-TAMRA can be conjugated to CAR-Y via click chemistry, enabling the visualization of CAR-Y’s subcellular localization. Previous studies have demonstrated that carabrone localizes to the mitochondria of *G. tritici* [16,17]. Based on these observations, the in vivo distribution of CAR-Y in *G. tritici* was analyzed using Mito-Tracker Green. After 4-hour treatment with 50 μM CAR-Y, the probe localized primarily to mitochondria (colocalized with Mito-Tracker Green and 5-TAMRA). However, at higher concentrations, CAR-Y spread to other organelles beyond mitochondria (Fig 3E). Meanwhile, elevated CAR-Y concentrations induced mycelial swelling, mitochondrial swelling, and vacuolization. Further analysis of mitochondrial protein labeling revealed that CAR-Y preferentially labeled proteins at 50 kDa and 60 kDa (Figs 3F and S3E). Therefore, 100 μM CAR-Y was selected for subsequent protein enrichment experiments.

CAR-Y-binding proteins were enriched separately from the total protein and mitochondrial protein fractions of *G. tritici*, followed by mass spectrometry identification and intersection analysis, which identified 145 carabrone-binding proteins (Figs 3G and S3F). Subcellular localization analysis revealed that 56 of these proteins are localized in the mitochondria, and they were thus identified as potential target proteins of carabrone (Fig 3G and S1 Table). GO and KEGG enrichment analysis of the 56 potential target proteins revealed their involvement in electron transfer activity and NAD-related oxidoreductase activity (Fig 3H); additionally, the TCA cycle, pyruvate metabolism, and OXPHOS pathways were significantly enriched, which is consistent with the downregulated gene clusters in the transcriptome analysis (Figs 3I and 1D). Further analysis revealed that 12 of the proteins, the highest proportion, utilize NAD^+^ and NADH as cofactors (Fig 3J). Strikingly, this finding aligns perfectly with the previously observed disruption of NAD^+^/NADH homeostasis by carabrone. This provides strong grounds to hypothesize that carabrone binds to NAD-related proteins to modulate NAD^+^/NADH homeostasis.

### The mitochondrial respiratory chain is a potential target of carabrone’s antifungal activity

**Fig 4:**
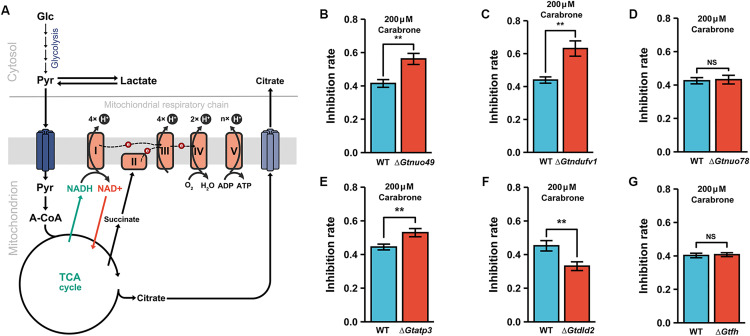
Screening of carabrone target proteins. **(A)** Schematic illustration of mitochondrial NAD⁺/NADH balance maintenance. **(B)** Sensitivity of Δ*Gtnuo49* silenced strains to carabrone. **(C)** Sensitivity of Δ*Gtndufv1* silenced strains to carabrone. **(D)** Sensitivity of Δ*Gtnuo78* silenced strains to carabrone. **(E)** Sensitivity of Δ*Gtatp3* silenced strains to carabrone. **(F)** Sensitivity of Δ*Gtdld2* silenced strains to carabrone. **(G)** Sensitivity of Δ*Gtfh* silenced strains to carabrone. The concentration of carabrone is 200 μM. Data are mean ± SD of *n* = 3 biologically independent experiments. Statistical significance was determined by one-way ANOVA with Tukey’s post hoc test (*P* < 0.05).

The mitochondrial respiratory chain (especially complex I) and the TCA cycle are key regulators of mitochondrial NAD^+^/NADH homeostasis (Fig 4A). The mitochondrial respiratory chain can convert NADH derived from the TCA cycle into NAD^+^, which then re-enters the TCA cycle to provide energy for cellular processes [36]. Additionally, mitochondrial D-lactate dehydrogenase also participates in NAD^+^/NADH metabolism [37]. Under conditions of respiratory inhibition, pyruvate can regenerate NAD^+^ via lactate dehydrogenase, enabling aspartate synthesis to support cell proliferation [38,39]. Based on this, we selected potential target proteins (from ABPP) of carabrone, including subunits *Gtnuo49*, *Gtndufv1*, and *Gtnuo78* of respiratory complex I, subunit *Gtatp3* of respiratory complex V, D-lactate dehydrogenase 2 (*Gtdld2*), and fumarate hydratase (*Gtfh*), to construct silencing mutants for validating changes in sensitivity to carabrone (S1 Table and S4A Fig). qPCR analysis confirmed that the silencing efficiency of the mutants reached over 60% (S4B Fig). Sensitivity assays to carabrone revealed that the complex I mutants, *∆Gtnuo49* and *∆Gtndufv1*, exhibited a significant increase in sensitivity to carabrone (Figs 4B, S4C, 4C and S4D). However, no obvious change in sensitivity was observed for *∆Gtnuo78*, possibly due to its relatively lower silencing efficiency (Figs 4D and S4E). Similarly, the complex V mutant *∆Gtatp3* also showed a significant increase in sensitivity to carabrone (Figs 4E and S4F). These results further confirm that carabrone exerts a substantial impact on the mitochondrial respiratory chain of *G. tritici*. In contrast, the sensitivity of *∆Gtdld2* to carabrone was significantly reduced, the underlying mechanism requires further investigation (Figs 4F and S4G). D-lactate dehydrogenase 2 is localized to the mitochondria and catalyzes the reversible conversion of D-lactate to pyruvate, coupled with the interconversion of NAD⁺ and NADH (Both NAD⁺-dependent and NAD⁺-independent isoforms exist) [40,41]. Nonetheless, no significant change in sensitivity to carabrone was observed for *∆Gtfh* (Figs 4G and S4H). As the TCA cycle represents the primary pathway for NAD^+^ consumption, its activity is regulated by the mitochondrial respiratory chain. These findings suggest that the mitochondrial respiratory chain is a potential target of carabrone’s antifungal activity, and carabrone may disrupt NAD^+^/NADH homeostasis by inhibiting the mitochondrial respiratory chain.

### Carabrone affects the ETC rather than the OXPHOS

**Fig 5:**
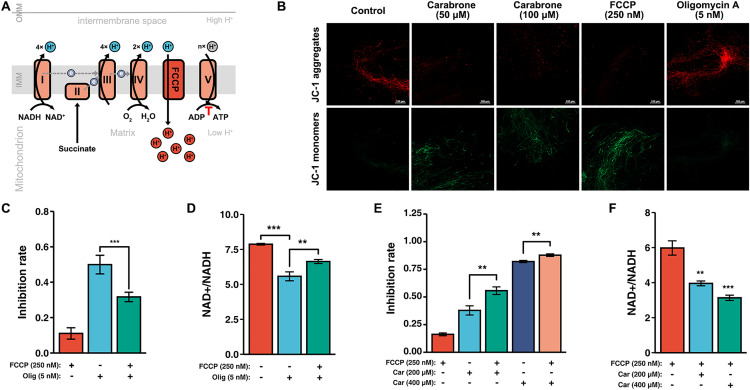
Effect of carabrone on mitochondrial respiratory chain complex V (ATP synthase). **(A)** Schematic illustration of OXPHOS and its uncoupling mechanism from the electron transport chain (ETC). **(B)** Measurement of mitochondrial membrane potential (MMP, Δ*Ψ*m) in *G. tritici*, treatment by carabrone (50 and 100 μM), FCCP (250 nM) and oligomycin A (5 nM), JC-1 monomers (green) indicate decreased MMP, JC-1 aggregates (red) indicate increased MMP, Bar = 100 μm. **(C)** Effect of FCCP (250 nM) on the antifungal activity of oligomycin A (5 nM). **(D)** NAD^+^/NADH ratio in *G. tritici* after FCCP (250 nM) and oligomycin A (5 nM) treatment. **(E)** Effect of FCCP (250 nM) on the antifungal activity of carabrone (200 and 400 μM). **(F)** NAD^+^/NADH ratio in *G. tritici* after FCCP (250 nM) and carabrone (200 and 400 μM) treatment. Data are mean ± SD of *n* = 3 biologically independent experiments. Statistical significance was determined by one-way ANOVA with Tukey’s post hoc test (*P* < 0.05).

The mitochondrial respiratory chain, composed of the ETC and OXPHOS, is a critical pathway for regulating NAD^+^/NADH homeostasis. As the starting point of the ETC, complex I directly influences NAD^+^ synthesis, while the inhibition of ETC and OXPHOS can also indirectly affect NAD^+^/NADH homeostasis [30,39]. Therefore, distinguishing the effects of carabrone on the mitochondrial respiratory chain is key to identifying its target. We hypothesized that the impact of carabrone on NAD^+^/NADH homeostasis results from the inhibition of complex V, which would accordingly lead to an increase in mitochondrial membrane potential (MMP) (hyperpolarization) (Fig 5A). However, following carabrone treatment, the mitochondrial membrane potential of *G. tritici* was depolarized rather than hyperpolarized (Fig 5B). FCCP, as an uncoupler of OXPHOS, can bypass complex V to transfer protons generated by the ETC back into the mitochondrial matrix (Fig 5A). As expected, co-treatment with FCCP and oligomycin A markedly decreased the antifungal activity of oligomycin A (Fig 5C). Meanwhile, FCCP partially restored the NAD^+^/NADH ratio in *G. tritici* treat by oligomycin A (Fig 5D). Therefore, FCCP can alleviate the hyperpolarization of the MMP caused by the inhibition of complex V, thereby restoring the ETC. In contrast, FCCP did not reduce the antifungal activity of carabrone but instead significantly enhanced it (Fig 5E). Moreover, FCCP failed to restore the NAD^+^/NADH homeostasis disrupted by carabrone (Fig 5F). In conclusion, it is evident that carabrone affects the ETC rather than OXPHOS, and complex V is not a target protein of carabrone.

### Carabrone inhibits mitochondrial respiratory chain complex I to modulate NAD^+^/NADH homeostasis

**Fig 6:**
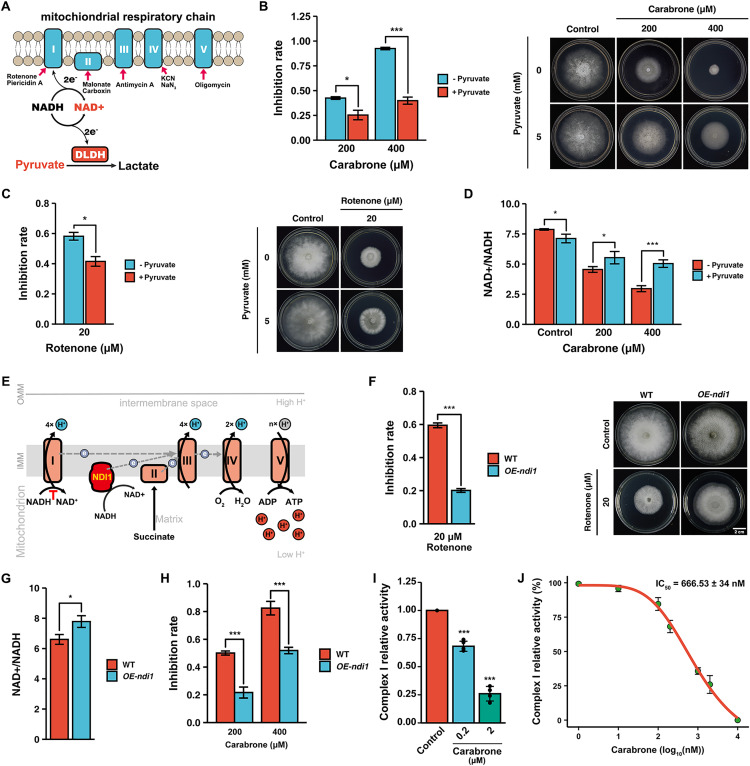
Validation of carabrone targeting mitochondrial respiratory chain complex I in *G. tritici.* **(A)** Schematic illustration of the mechanism by which pyruvate converts NADH to NAD⁺ via D-lactate dehydrogenase to support complex I function. **(B)** Modulation of carabrone’s (200 and 400 μM) antifungal activity by pyruvate (5 mM) supplementation. **(C)** Modulation of rotenone’s (20 μM) antifungal activity by pyruvate (5 mM) supplementation. **(D)** NAD^+^/NADH ratio in *G. tritici* after carabrone (200 and 400 μM) and pyruvate (5 mM) treatment. **(E)** Schematic illustration of the mechanism by which *ndi1* overexpression supports mitochondrial complex I function. **(F)** Sensitivity of *ndi1* overexpression strain to rotenone (20 μM). **(G)** NAD^+^/NADH ratio in *ndi1* overexpression strain of *G. tritici*. **(H)** Sensitivity of *ndi1* overexpression strain to carabrone (200 and 400 μM). (**I**) and **(J)** Enzymatic activity assay of mitochondrial respiratory chain complex I in *G. tritici* treated with carabrone. Data are mean ± SD of *n* = 3 biologically independent experiments. Statistical significance was determined by one-way ANOVA with Tukey’s post hoc test (*P* < 0.05).

It has been reported that pyruvate can serve as an electron acceptor for NADH to regenerate NAD^+^ and enable aspartate synthesis, thereby supporting cell proliferation when the ETC is inhibited (Fig 6A) [37–39]. As expected, addition of pyruvate to PDA medium significantly reduced the sensitivity of *G. tritici* to carabrone (Fig 6B). Similarly, the inhibition rate of the complex I inhibitor rotenone on *G. tritici* was also significantly decreased in the presence of pyruvate (Fig 6C). Further experiments confirmed that pyruvate could partially restore the NAD^+^/NADH balance disrupted by carabrone (Fig 6D). These results preliminarily suggest that carabrone may exert its antifungal activity by targeting complex I, thereby disrupting NAD^+^/NADH homeostasis.

Mitochondrial respiratory chain complex I is a proton-pumping NADH dehydrogenase (NDH-1), while non-proton-pumping NADH dehydrogenases (NDH-2) exist in plants, fungi, and bacteria [42–44]. In *Saccharomyces cerevisiae*, complex I is absent, and NDH-2 enzymes (including Ndi1 and Nde1) fulfill the electron transfer function of complex I by converting NADH to NAD^+^ [45,46]. Notably, Ndi1 faces the mitochondrial matrix and plays a greater role than outer-membrane Nde1 in regulating NAD⁺/NADH homeostasis [47]. Among these, Ndi1 serves as the primary entry point for the mitochondrial respiratory chain in *S. cerevisiae*. Therefore, to verify the targeting effect of carabrone on complex I, we planned to overexpress NDH-2 in *G. tritici* (Fig 6E). However, there are few reports on the functionality of NDH-2 in phytopathogenic fungi, and the specific roles of related genes remain unclear. Consequently, we successfully overexpressed the *ndi1* gene from *S. cerevisiae* s288c in *G. tritici* (S6A and S6B Fig). After overexpressing *ndi1* in *G. tritici* (*OE-ndi1*), its sensitivity to rotenone was significantly reduced (Fig 6F). Meanwhile, the NAD^+^/NADH ratio in the *OE-ndi1* strain slightly increased (Fig 6G). These results indicate that the overexpression of *ndi1* in *G. tritici* can partially compensate for the function of complex I by remodeling NAD^+^/NADH homeostasis (Fig 6E). Similar to rotenone, the sensitivity of the *OE-ndi1* strain to carabrone was also markedly decreased (Figs 6H and S6C). This suggests that complex I is one of the antifungal targets of carabrone. Enzyme activity assays revealed that 200 nM carabrone significantly inhibited the activity of complex I in *G. tritici* (with an inhibition rate of 32%), and its IC_50_ was determined to be 666.53 nM (Fig 6I and 6J). Thus, it can be concluded that carabrone exhibits strong inhibitory activity against complex I in *G. tritici*.

Previous studies have also indicated that carabrone exhibits inhibitory activity against ETC complex III [17,18]. To verify this, the sensitivity of the *OE-ndi1* strain to complex III inhibitors was assessed. However, no significant change in sensitivity to azoxystrobin was observed in the *OE-ndi1* strain (S6D Fig). This demonstrates that NDI1 can partially compensate for the function of complex I, thereby altering the sensitivity to complex I inhibitors, but it does not affect the sensitivity to other ETC inhibitors. Based on these findings, it can be concluded that complex I is the primary antifungal target of carabrone, and carabrone exerts its antifungal effects by targeting complex I to disrupt NAD^+^/NADH homeostasis.

## Discussion

Chemical control remains a critical strategy for plant disease management. However, excessive and irrational application of fungicides has weakened the positive correlation between disease control efficacy and cost. Rational use of existing agents is essential, but uncovering novel antifungal targets and developing next-generation fungicides represent a sustainable solution. Natural products, with their structural diversity, not only provide novel scaffolds for fungicide discovery but also offer unprecedented opportunities for identifying new modes of action.

This study demonstrates that the SL compound carabrone exerts antifungal activity against *G. tritici* by targeting mitochondrial respiratory chain complex I and disrupting NAD⁺/NADH homeostasis. Previous studies revealed carabrone’s broad-spectrum inhibitory activity against plant pathogenic fungi, with the strongest efficacy observed against *G. tritici* [13]. Pot experiments confirmed carabrone’s strong efficacy against wheat take-all and powdery mildew (500 mg/L comparable to 150 mg/L triadimefon), and possesses systemic translocation activity from roots to leaves [48,49]. Structural modification and structure-activity relationship (SAR) analyses identified *α*-methylene-*γ*-butyrolactone as the critical pharmacophore, prompting extensive derivative synthesis [14,50,51]. Initial mechanistic investigations showed that carabrone localizes to *G. tritici* mitochondria, inducing mitochondrial dysfunction, ROS burst, and subsequent apoptosis [15,16]. Enzyme activity assays also showed that carabrone exerts differential effects on mitochondrial respiratory chain complexes, with the most significant inhibition observed against complex I, III, I+III, and II+III activities [18]. However, the direct molecular target remained elusive. The *α*-methylene-*γ*-butyrolactone moiety forms irreversible covalent bonds with cysteine residues in proteins, introducing potential off-target effects during prolonged treatment and complicating target identification. To address this, time-series transcriptomic profiling of *G. tritici* during early carabrone exposure (1–4 h) was performed. Results confirmed significant perturbation of the OXPHOS pathway (Fig 1), consistent with prior findings [18]. Further analysis revealed a strong association between carabrone treatment and the nicotinate/nicotinamide metabolism pathway (Fig 2A and 2B), a key regulator of cellular NAD⁺ biosynthesis and NAD⁺/NADH balance. As essential cofactors in central metabolic pathways (e.g., glycolysis, fatty acid oxidation, TCA cycle, and OXPHOS), NAD⁺ and NADH redox imbalance disrupts metabolic flux, leading to systemic cellular dysfunction [31]. Maintaining optimal NAD⁺/NADH ratios is thus critical for redox homeostasis and cellular viability. Experimental validation demonstrated that carabrone destabilizes this equilibrium, and enhancing NAD⁺ synthesis through orthogonal pathways significantly reduces *G. tritici* sensitivity to carabrone, establishing a direct link between NAD⁺ dynamics and its antifungal mechanism (Fig 2).

ABPP integrates activity-based probes (ABPs) with proteomics to identify molecular targets of bioactive small molecules [52]. Alkyne-modified ABPs enable click chemistry-mediated conjugation with reporter groups, facilitating protein labeling, in situ imaging, and enrichment [53]. Combining in *vivo* and in *vitro* labeling enhances the reliability of target identification. Based on this approach, we synthesized an alkyne-tagged carabrone probe (CAR-Y), which was rigorously validated for its efficacy in capturing carabrone targets through both cellular and cell-free assays (Figs 3 and S3). Fluorescently labeled CAR-Y localized predominantly to *G. tritici* mitochondria, consistent with prior subcellular localization studies [16,17]. Guided by this observation, CAR-Y-bound proteins were enriched from total and mitochondrial fractions of *G. tritici*, identifying 56 putative target proteins. Notably, quantitative proteomics was not employed in this study, precluding direct target prioritization via abundance profiling. However, subcellular localization and NAD⁺/NADH ratio analyses provided critical functional context, with many candidate targets mechanistically linked to NAD⁺/NADH homeostasis. Ultimately, through gene silencing, drug sensitivity assays, and physiological and biochemical analyses, mitochondrial respiratory chain complex I, a key regulator of NAD⁺/NADH homeostasis, was identified as one of the target proteins of carabrone. (Figs 4–6).

Mitochondrial respiratory chain complex I serves as a core component of the ETC and a key regulator of NAD⁺/NADH homeostasis in plant pathogenic fungi. Targeting this complex offers a promising strategy to combat escalating fungicide resistance. However, its structural complexity and the lack of complete structural data in plant pathogenic fungi hinder structure-based rational design of fungicides. Identifying lead compounds that target complex I represent an effective current approach. SLs exhibit broad biological activities, including antitumor, antimalarial, antifungal, and antibacterial effects, underscoring their potential as lead scaffolds [54–56]. Parthenolide, for instance, demonstrates potent inhibition against *Xanthomonas oryzae* pv. *oryzae*, with its interaction with the NuoF subunit of complex I validated through pull-down assays, binding affinity measurements, and crystallography [57–59]. This study reveals that carabrone, another SL, targets complex I in *G. tritici*, highlighting the therapeutic potential of carabrone and parthenolide as leads for developing complex I-targeted fungicides. Notably, our group has previously synthesized a series of carabrone derivatives, yielding highly active compounds that show promise as complex I-targeting fungicides [13,14,50,51]. Fungal complex I comprises at least 45 subunits, requiring coordinated interactions for functionality; thus, analyzing carabrone’s binding to individual subunits fails to fully elucidate its mechanism. For example, rotenone engages complex I at three distinct sites, including two decylubiquinone-binding pockets, while metformin binds simultaneously to the quinone-binding channel and a membrane-proximal pocket [60,61]. These precedents suggest carabrone’s interaction with *G. tritici* complex I likely involves a complex mode of action requiring systematic investigation. Studies on complex I function in plant pathogenic fungi remain scarce, and validated examples of compound-complex I interactions are notably absent, further complicating drug development. By leveraging NAD⁺/NADH ratio perturbations as a functional readout, this study first demonstrated that carabrone’s antifungal activity is independent of complex V, thereby attributing its primary impact to the ETC (Fig 5). Through exogenous pyruvate supplementation, heterologous overexpression of *S. cerevisiae ndi1* (a single-subunit NADH dehydrogenase), and physiological/biochemical validation, carabrone’s disruption of NAD⁺/NADH homeostasis was mechanistically linked to its targeting of respiratory complex I (Fig 6A-H). This direct interaction was further confirmed via *in vitro* enzymatic assays, which established complex I as carabrone’s molecular target (Fig 6I and 6J). Moreover, overexpression of *Scndi1* did not alter sensitivity to the complex III inhibitor azoxystrobin. Carabrone’s reported complex III effect is likely a downstream consequence of complex I inhibition impairing electron transfer, not direct (CoQ cycle disruption) [17,18,62]. Notably, the utility of *Scndi1* overexpression in plant pathogens provides a robust experimental framework for distinguishing mitochondrial ETC inhibitors.

Notably, alternative NADH dehydrogenases—functionally redundant with mitochondrial complex I—are broadly present in fungi and plants [44,63]. These enzymes mediate rotenone-insensitive oxidation of extramitochondrial NADH and perform parallel functions to complex I [45]. As single- or oligomeric-subunit flavoproteins with multiple isoforms, they catalyze the same NADH oxidation reaction as complex I but do not contribute to proton translocation required for ATP synthesis [42,44]. In *Neurospora crassa*, the alternative NADH dehydrogenase NDI1 complements complex I and is essential for spore germination [64]. In mammals, NDI1 overexpression rescues complex I deficiency, restoring mitochondrial function and ameliorating associated defects, including aging and Parkinson’s disease [65,66]. However, the specific composition of alternative NADH dehydrogenases in plant pathogenic fungi and their role in regulating complex I activity remain poorly understood. This knowledge gap likely hinders the development of complex I-targeted fungicides. In this study, heterologous overexpression of *S. cerevisiae ndi1* in *G. tritici* demonstrated that NDI1 modulates NAD⁺/NADH homeostasis and alters fungal sensitivity to both rotenone and carabrone. These findings demonstrated yeast NDI1 as a valuable tool for validating complex I-targeting fungicides in crop pathogens. Concurrently, elucidating the functional mechanisms and physiological significance of alternative NADH dehydrogenases in phytopathogens is critical for advancing mitochondrial-targeted antifungal strategies.

In summary, the SL carabrone isolated from *C. macrocephalum* exhibits potent antifungal activity against *G. tritici* and other phytopathogens. Integrative analyses of time-series transcriptomics, ABPP, biochemical assays, and functional validation revealed that carabrone targets mitochondrial respiratory chain complex I in *G. tritici*, disrupting NAD⁺/NADH homeostasis and triggering ROS accumulation, which ultimately induces apoptosis and inhibits pathogen growth. This study represents the first mechanistic characterization of carabrone’s antifungal target, providing both a structural scaffold for developing novel complex I-targeted fungicides and a paradigm for validating mitochondrial complex I as a druggable antifungal target.

## Materials and methods

### Chemicals, fungal strains, bacterial strains, plasmids, and growth conditions

Carabrone (purity ≥ 95%) was isolated and obtained by our research group from *C. macrocephalum*. Rotenone (CAS: 83-79-4; purity ≥ 95%), oligomycin A (CAS: 579-13-5; purity ≥ 99%), FCCP (CAS: 370-86-5; purity ≥ 95%), azoxystrobin (CAS: 131860-33-8; purity ≥ 98%) were purchased from Macklin Co., Ltd (Shanghai, China). *G. tritici* was the primary strain used for activity assays, target identification, mutant construction, and physiological/biochemical analyses. *Escherichia coli* DH5α was used for vector construction in this study*. Agrobacterium tumefaciens* EHA105 was used for infecting *G. tritici* to perform genetic transformation. *G. tritici* was cultured at 25°C on PDA and PDB media (Cat: 254920, Becton, Dickinson and Company, USA; pH 5.1). *E. coli* DH5α (37°C) and *Agrobacterium tumefaciens* EHA105 (28°C) were cultured in LB medium.

### Antifungal activity tests in *vitro*

The inhibitory activity of carabrone against *G. tritici* was determined using the mycelial growth rate method [67]. Briefly, PDA medium was mixed with different concentrations of carabrone, CAR-Y, rotenone, or oligomycin A and then poured into petri dishes. PDA medium containing 0.25% DMSO served as the blank control. For experiments involving the addition of NMN, durohydroquinone, or pyruvate, the controls consisted of PDA medium supplemented only with the respective compound, while the treatments included both the compound and carabrone/rotenone. A 5-mm mycelial plug was placed at the center of each petri dish, which was then incubated at 25°C for 5–7 days. Three replicates were performed for each parallel experiment. The rate of mycelial growth inhibition was calculated using the following formula


$$\text{Mycelial growth inhibition ratio}=\;\left[\frac{\left(dc-dt\right)}{\left(dc-\text{5}\text{ mm}\right)}\right]\times \text{100}\%$$

where *dc* and *dt* represent the mean diameters in the control and treatment groups, respectively.

### RNA isolation, RNA-seq and quantitative real-time PCR

A mycelial plug was taken from the edge of the *G. tritici* and placed in PDB medium, followed by incubation at 25°C on a shaking incubator for 3 days. Subsequently, 200 μM carabrone was added, and the culture was further incubated for 1, 2, or 4 hours. The addition of 0.25% DMSO served as the blank control (without shaking). Total RNA from the samples was extracted using the TRIzol method following the product’s operational protocol (Cat: R0016, Beyotime Biotechnology, Shanghai, China). Subsequently, the RNA samples were sent to Beijing Novogene Technology Co., Ltd. (Beijing, China) for RNA-seq analysis, the data of gene expression were shown in S2 Table. DEGs were identified based on |log_2_(FoldChange)| ≥ 2 and *P* value < 0.05. GO and KEGG enrichment analyses of the DEGs were performed using clusterProfiler (version 4.4.4), with Adjusted *p* < 0.05 considered as significantly enriched [68].

cDNAs were synthesized using a reverse transcription kit (Cat: R212-01, Vazyme, Nanjing, China). Quantitative real-time PCR was performed using the ChamQ SYBR qPCR Master Mix (Without ROX) kit (Cat: Q321-02, Vazyme, Nanjing, China), with 18sRNA serving as the internal reference gene [17]. Data collection and analysis were performed using the 2^-ΔΔCT^ method on the Applied Biosystems QuantStudio 5 system (Thermo Fisher Scientific Inc., USA). The relevant quantitative primers are listed in S3 Table.

### ATP content assay

The Enhanced ATP Assay Kit (Cat: S0027, Beyotime Biotechnology, Shanghai, China) was used to measure ATP levels in *G. tritici*. Mycelia cultured for 3 days under shaking conditions were treated with carabrone, rotenone, or oligomycin A for 6 h, then harvested, lysed, and sonicated. After centrifugation at 4°C, the supernatant was collected. A 20 μL sample was mixed with ATP detection reagent, and luminescence (RLU) was measured. ATP concentrations were calculated using a standard curve and normalized to protein content to account for sample variability.

### NAD^+^/NADH ratio assay

The levels of NAD^+^ and NADH in the mycelial lysates were measured using a WST-8 colorimetric assay (Cat: S0176S, Beyotime Biotechnology, Shanghai, China), and the NAD^+^/NADH ratio was subsequently calculated [69]. Briefly, mycelia were treated, ground, lysed, and centrifuged to prepare samples. Samples (20 μL) were mixed with alcohol dehydrogenase solution (90 μL), incubated at 37°C for 10 min, and reacted with 10 μL chromogenic reagent. Absorbance was measured at 450 nm to calculate total NAD^+^ and NADH using an NADH standard curve. NADH was determined after heat treatment at 60°C for 30 min to decompose NAD^+^, enabling calculation of NAD^+^ content and the NAD^+^/NADH ratio. Protein concentrations were measured using the BCA method to calculate the total or individual levels of NAD^+^ and NADH per unit of protein.

### Protein labeling in *vivo* and in *vitro*

To identify the antifungal target of carabrone, we synthesized a carabrone-derived alkyne probe (CAR-Y, S3A Fig and S1 Text) and analyzed its potential antifungal targets using the ABPP [70]. First, *G. tritici* was cultured in PDB medium for 3 days under shaking conditions. CAR-Y was added to the mycelia with or without carabrone, followed by an additional 4 h incubation. The mycelia were then washed with phosphate-buffered saline (PBS), collected, and lysed using a total protein extraction reagent (Cat: R0030, Solarbio Life Sciences, Beijing, China). Total protein extract (2 mg/mL in PBS) was reacted with click chemistry reagents (0.25 M NaASC, 50 mM CuSO₄, 50 mM BTTAA, 10 mM Biotin-PEG_3_-N_3_) at room temperature for 1 h with shaking. Proteins were precipitated with ice-cold acetone, resuspended in 1 × loading buffer, and subjected to streptavidin blotting to assess CAR-Y labeling efficiency on *G. tritici* total proteins.

Mitochondria were isolated from *G. tritici* mycelia via differential centrifugation [18]. Mycelia were homogenized in mitochondrial extraction buffer, centrifuged at 1,500 × *g* (4°C, 10 min) after resuspension, and the supernatant was collected. Following centrifugation at 10,000 × *g* (4°C, 20 min), the pellet was washed with BSA-free mitochondrial extraction buffer and recentrifuged under identical conditions. The final pellet (crude mitochondrial extract) was retained for protein extraction and subsequent CAR-Y-labeled protein detection/enrichment.

### Protein labeling for in situ imaging

*G. tritici* mycelia were treated with varying concentrations of CAR-Y and subsequently fixed with 4% paraformaldehyde in PBS for 60 mins at room temperature. Following fixation, the mycelia were permeabilized by enzymatic digestion with lysing enzymes for 30 min. For mitochondrial staining, the mycelia were incubated with Mito-Tracker Green at 37°C in the dark for 30 min. After three washes with PBS to remove unbound probe, click chemistry reaction mixtures (0.25 M NaASC, 50 mM CuSO_4_, 50 mM BTTAA, and 10 mM 5-TAMRA-PEG_3_-Azide) were added. The samples were incubated in the dark at room temperature with gentle agitation for 1 hour to facilitate fluorophore conjugation. Excess reagents were removed by extensive PBS washing, and the subcellular localization of CAR-Y was visualized using a confocal laser scanning microscope (CLSM) equipped with appropriate filter sets for TAMRA (546/575 nm) and Mito-Tracker Green (490/516 nm).

### ABPP-based identification of targets

For enrichment, CAR-Y labeled proteins (dissolved in PBS with 1.5% SDS) were incubated with streptavidin magnetic beads overnight at 4°C. Beads were successively washed with 6 M urea, 1% SDS/PBS, and PBS (3 × each), then denatured in 1 × loading buffer at 95°C for 10 min. Enriched proteins were resolved by SDS-PAGE and visualized by Coomassie Brilliant Blue staining. Target bands were excised from SDS-PAGE gel, washed with 50% CH_3_CN/100 mM NH_4_HCO_3_, and dehydrated with acetonitrile. Disulfide bonds were reduced with 10 mM DTT/50 mM NH_4_HCO_3_ and alkylated with 60 mM IAM/50 mM NH_4_HCO_3_. After further dehydration, trypsin was added for digestion at 37°C for 12 h. The digested peptides were collected, desalted, and analyzed using LC-MS/MS with settings: Ion Source Type: NSI, Orbitrap Resolution: 60,000, Scan Range: 375–1500, AGC Target: 5.0e4. Data were processed using Proteome Discoverer and matched with the *G. tritici* protein database to identify carabrone-binding proteins.

### Construction of silencing and overexpression mutant strains

Silencing mutants were constructed via *PEG/CaCl₂*-mediated protoplast transformation using pSilent-Dual1 (Ampicillin/G418 resistance) [63]. The *OE-ndi1* (*ndi1* from *S. cerevisiae* s288c; NDI1 is subcellularly localized to the mitochondria and faces the mitochondrial matrix.) strain was generated through *Agrobacterium*-mediated transformation using pCAMBIA1303-gpdA-Trpc-Hygro (kanamycin/hygromycin B resistance) [71]. Primers for vector construction are listed in S2 Table. All strains were verified by PCR, sequencing and RT-qPCR. All antibiotics used in this study were purchased from Coolaber Life Sciences, Beijing, China.

### Fluorescence staining

The intracellular superoxide anion (O_2_^.-^) level was detected using the fluorescent probe Dihydroethidium (DHE) (Cat: S0064S, Beyotime Biotechnology, Shanghai, China), which is dehydrogenated in the presence of superoxide anions to produce red fluorescence [72]. After treating *G. tritici* mycelia with carabrone or rotenone for 4 h, the mycelia were washed with PBS and incubated with 10 μM DHE at 37°C for 30 min. Subsequently, the mycelia were thoroughly washed three times with PBS and observed under a confocal laser scanning microscope (518/610 nm).

The MMP of *G. tritici* was detected using the JC-1 fluorescent probe (Cat: C2006, Beyotime Biotechnology, Shanghai, China), which emits red fluorescence when the MMP is high and green fluorescence when it is low [73]. The probe treatment process was as described above. After thorough washing, the mycelial fluorescence was observed under a confocal laser scanning microscope (490/530 nm, 525/590 nm).

### Mitochondrial respiratory chain complex I activity assay

Isolation of mitochondrial inner membranes fractions. Purified *G. tritici* mitochondria were homogenized and supplemented with 150 mM KCl. Centrifugation at 50,000 × *g* for 45 min isolated the inner membrane fraction, which was resuspended in buffer M (20 mM HEPES, 40 mM NaCl, 1 mM EDTA, 10% v/v glycerol, 2 mM DTT, 0.002% PMSF, pH 7.4) and manually homogenized. A second centrifugation (50,000 × *g*, 45 min) further purified the membrane pellet, which was resuspended in buffer M to 5 mg/mL protein and stored on ice for analysis.

Complex I activity assay. The enzymatic activity of complex I was measured according to the method described by Burger, with slight modifications [74]. Mitochondrial complex I activity was measured in a 96-well plate (200 µL/well). Mitochondrial membranes (10 μL) in buffer B (25 mM KCl, 25 mM MOPS, pH 7.4) were treated with 0.3 μM antimycin A (inhibits complex III), 0.2 mM KCN (inhibits complex IV), and 100 μM decylubiquinone. After adding carabrone (treatment) or DMSO (control), samples were incubated at 30°C for 10 min. The reaction was initiated with 0.2 mM NADH, and NADH oxidation was monitored at 340 nm (ε = 6.22 × 10³ L/mol/cm) every 30s for 10 min. Rotenone-sensitive activity was calculated by subtracting rotenone-insensitive activity (measured with 0.5 µM rotenone) from total activity. Calculation Formula:


$$\text{Enzyme activity }(\text{U}/\text{mg prot})=[\Delta A\times Va/(\varepsilon \times d)\times {\text{10}}^{\text{9}}]÷(Vs\times Cpr)÷T$$

*Va*: Total reaction volume (2 × 10^-4^ L); *ε*: Molar extinction coefficient of NADH (6.22 × 10^3^ L/mol/cm); *d*: Optical path length of the 96-well plate (0.6 cm); *Vs*: Volume of protein sample added (0.01 mL); *Cpr*: Protein concentration in the sample (mg/mL); 10^9^: Unit conversion factor (1 mol = 10^9^ nmol); *T*: Reaction time (10 min)

### Statistical analysis

All statistical analyses were performed using R (version 4.3.1). Data were processed with packages including *dplyr*, *ggplot2*, and *lme4*, and tested for normality (Shapiro-Wilk) and variance homogeneity (Levene’s test). Parametric (t-test/ANOVA) or non-parametric tests were applied based on data distribution. Mixed-effects models accounted for random effects, with post-hoc comparisons adjusted via Benjamini-Hochberg correction (*FDR *< 0.05). Graphs show mean ± SD, with significance defined as *p < 0.05* (*), *p < 0.01* (**), and *p < 0.001* (***).

## Supporting information

S1 Fig Time-series transcriptomic analysis of carabrone-treated *Gaeumannomyces tritici*, related to Fig 1. (A) The principal component analysis (PCA). (B) Heatmap of differentially expressed genes TPM value. (C) and (D) The number of DEGs compared to the control. (E) Gene ontology (GO) enrichment analysis of gene cluster 4 in total DEGs. (F) Heatmap of gene cluster 3 in the oxidative phosphorylation (OXPHOS) pathway.
(TIF)

S2 Fig Analysis of the correlation between NAD and the antifungal activity of carabrone, related to Fig 2. (A) Heatmap of gene expression clusters in cofactor biosynthesis pathway. (B) GO enrichment analysis of gene cluster 2 in cofactor biosynthesis pathway. (C) KEGG enrichment analysis of gene cluster 2 in cofactor biosynthesis pathway. (D) Modulation of carabrone’s (200 and 400 μM) antifungal activity by NMN (400 μM) supplementation. (E) Modulation of carabrone’s (400 μM) antifungal activity by duroquinone (10 and 40 μM) supplementation.
(TIF)

S3 Fig Identification of the antifungal target of carabrone by activity-based protein profiling (ABPP), related to Fig 3. (A) Synthetic scheme of the carabrone alkynyl probe (CAR-Y) for ABPP. (B) In *vitro* labeling of CAR-Y in total protein of *G. tritici*. (C) In *vitro* competitive labeling of CAR-Y in total protein of *G. tritici*. (D) In *vivo* labeling of CAR-Y time-dependent in *G. tritici*. (E) In *vitro* competitive labeling of CAR-Y in mitochondria protein of *G. tritici*. (F) Venn diagram analysis of carabrone-binding protein in *G. tritici*. CBB: coomassie brilliant blue.
(TIF)

S4 Fig Screening of carabrone target proteins, related to Fig 4. (A) PCR validation of gene-silenced mutants in *G. tritici*. (B) RT-qPCR validation of gene-silenced mutants in *G. tritici*. (C) Sensitivity of Δ*Gtnuo49* silenced strains to carabrone (200 μM). (D) Sensitivity of Δ*Gtndufv1* silenced strains to carabrone (200 μM). (E) Sensitivity of Δ*Gtnuo78* silenced strains to carabrone (200 μM). (F) Sensitivity of Δ*Gtatp3* silenced strains to carabrone (200 μM). (G) Sensitivity of Δ*Gtdld2* silenced strains to carabrone (200 μM). (H) Sensitivity of Δ*Gtfh* silenced strains to carabrone (200 μM). Data are mean ± SD of *n* = 3 biologically independent experiments. Statistical significance was determined by one-way ANOVA with Tukey’s post hoc test (*P* < 0.05).
(TIF)

S5 Fig Effect of FCCP (250 nM) on the antifungal activity of oligomycin A (5 nM) and carabrone (200 and 400 μM), related to Fig 5. (TIF)

S6 Fig Verification and phenotypic characterization of the *ndi1* overexpression strain in *G. tritici*, related to Fig 6. (A) PCR validation of the *ndi1* overexpression strain in *G. tritici*. (B) RT-qPCR validation of the *ndi1* overexpression strain in *G. tritici*. (C) Sensitivity of *ndi1* overexpression strain to carabrone (200 and 400 μM). (D) Sensitivity of *ndi1* overexpression strain to azoxystrobin (20 μM). Data are mean ± SD of *n* = 3 biologically independent experiments. Statistical significance was determined by one-way ANOVA with Tukey’s post hoc test (*P* < 0.05).
(TIF)

S1 Table The potential target proteins of carabrone in *G. tritici.* (XLSX)

S2 Table RNA-seq data (FPKM values) of *G. tritici* following carabrone treatment at 1, 2, and 4 hours. (XLSX)

S3 Table The primers used for plasmid constructions and RT-qPCR in this study. (XLSX)

S4 Table Raw data used in this study. (XLSX)

S1 Text Synthesis of the carabrone alkynyl probe (CAR-Y). (DOCX)
