# Supplementary figures and images for: Carabrone inhibits Gaeumannomyces tritici growth by targeting mitochondrial complex I and destabilizing NAD⁺/NADH homeostasis
Source: PLoS Pathog. 2025 Oct 3;21(10):e1013567. doi: 10.1371/journal.ppat.1013567 (PMC12510646; doi:10.1371/journal.ppat.1013567)

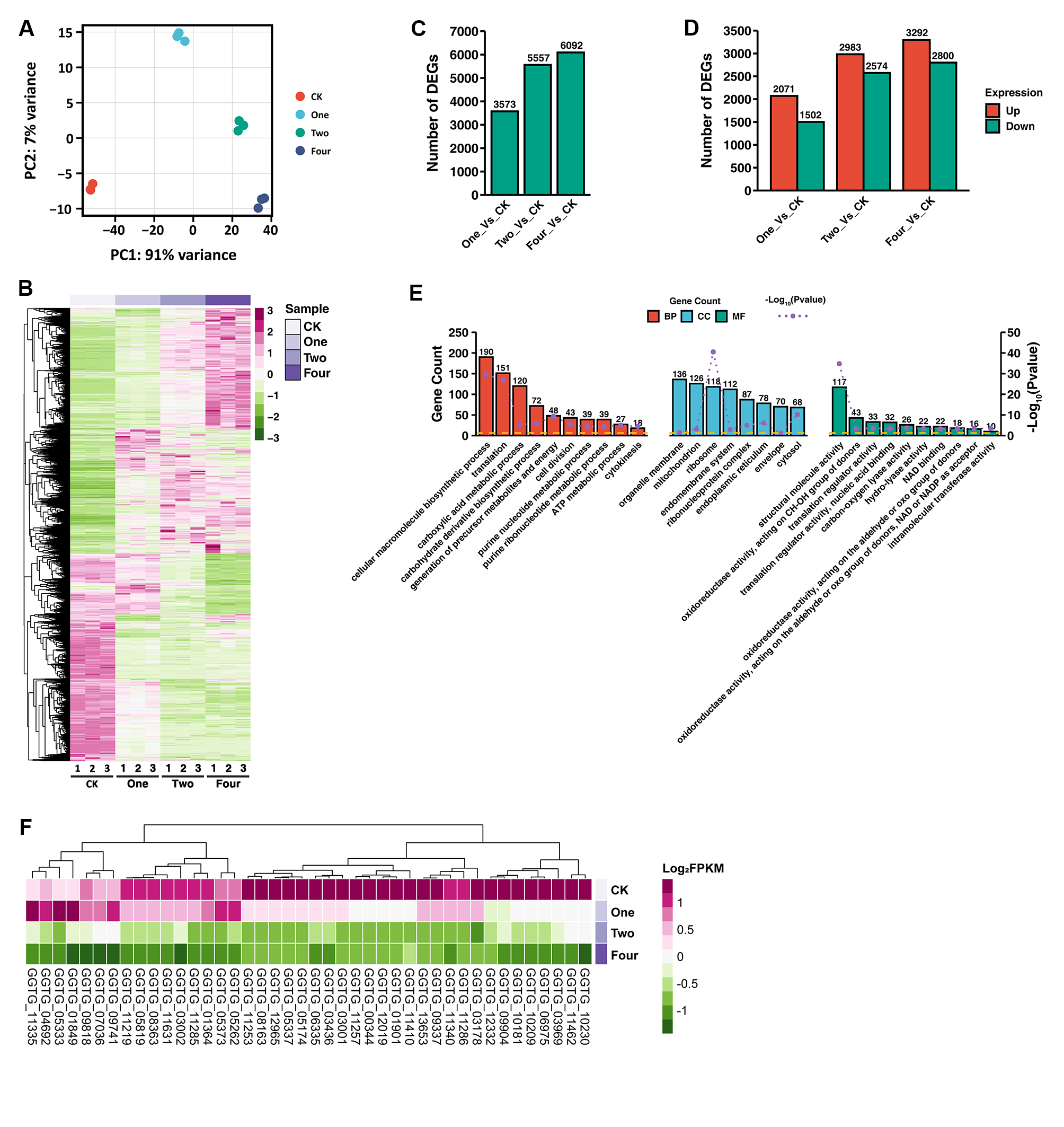

Supplement: S1 Fig — (A) The principal component analysis (PCA). (B) Heatmap of differentially expressed genes TPM value. (C) and (D) The number of DEGs compared to the control. (E) Gene ontology (GO) enrichment analysis of gene cluster 4 in total DEGs. (F) Heatmap of gene cluster 3 in the oxidative phosphorylation (OXPHOS) pathway. (TIF) [file ppat.1013567.s001.tif]

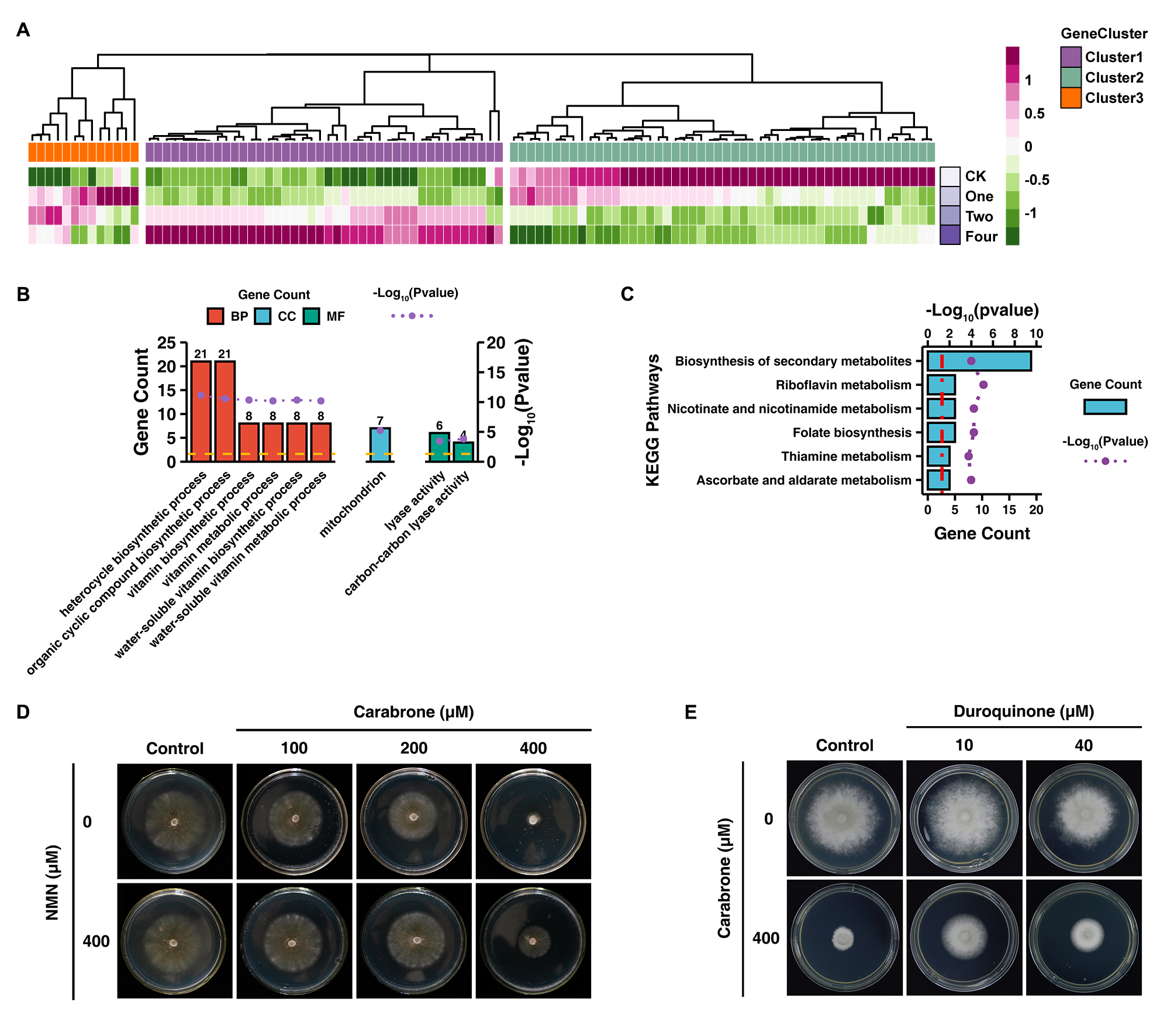

Supplement: S2 Fig — (A) Heatmap of gene expression clusters in cofactor biosynthesis pathway. (B) GO enrichment analysis of gene cluster 2 in cofactor biosynthesis pathway. (C) KEGG enrichment analysis of gene cluster 2 in cofactor biosynthesis pathway. (D) Modulation of carabrone’s (200 and 400 μM) antifungal activity by NMN (400 μM) supplementation. (E) Modulation of carabrone’s (400 μM) antifungal activity by duroquinone (10 and 40 μM) supplementation. (TIF) [file ppat.1013567.s002.tif]

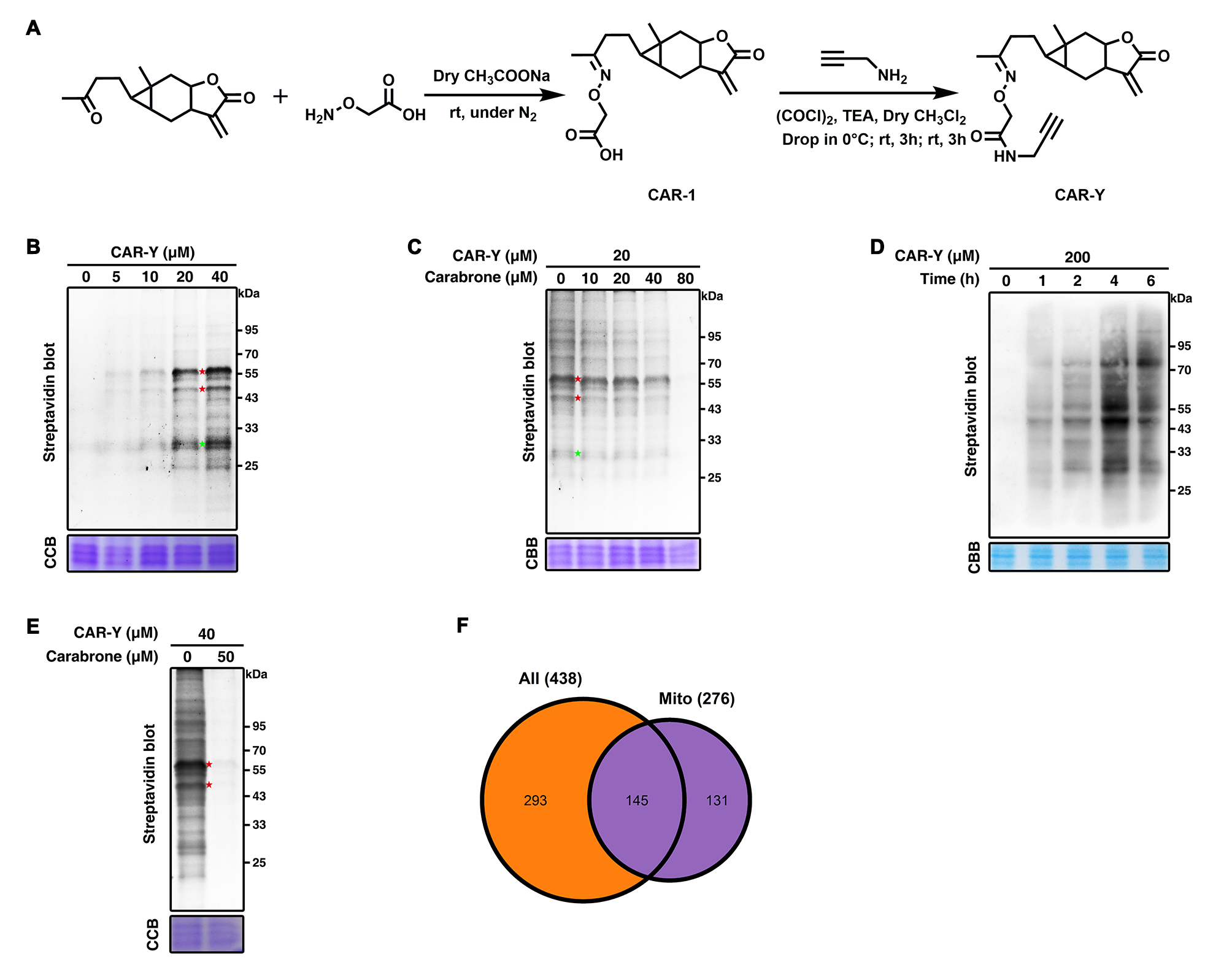

Supplement: S3 Fig — (A) Synthetic scheme of the carabrone alkynyl probe (CAR-Y) for ABPP. (B) In vitro labeling of CAR-Y in total protein of G. tritici. (C) In vitro competitive labeling of CAR-Y in total protein of G. tritici. (D) In vivo labeling of CAR-Y time-dependent in G. tritici. (E) In vitro competitive labeling of CAR-Y in mitochondria protein of G. tritici. (F) Venn diagram analysis of carabrone-binding protein in G. tritici. CBB: coomassie brilliant blue. (TIF) [file ppat.1013567.s003.tif]

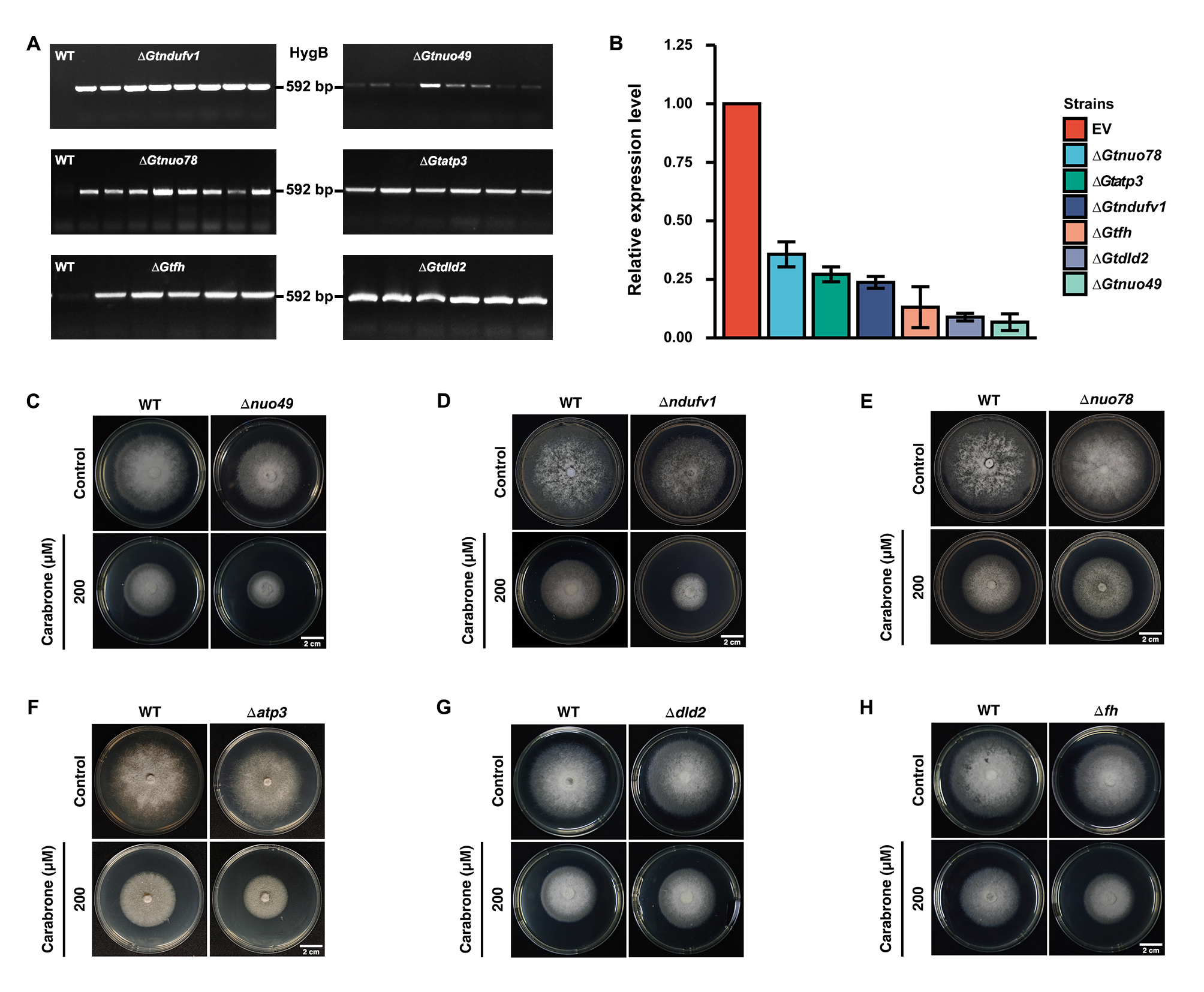

Supplement: S4 Fig — (A) PCR validation of gene-silenced mutants in G. tritici. (B) RT-qPCR validation of gene-silenced mutants in G. tritici. (C) Sensitivity of ΔGtnuo49 silenced strains to carabrone (200 μM). (D) Sensitivity of ΔGtndufv1 silenced strains to carabrone (200 μM). (E) Sensitivity of ΔGtnuo78 silenced strains to carabrone (200 μM). (F) Sensitivity of ΔGtatp3 silenced strains to carabrone (200 μM). (G) Sensitivity of ΔGtdld2 silenced strains to carabrone (200 μM). (H) Sensitivity of ΔGtfh silenced strains to carabrone (200 μM). Data are mean ± SD of n = 3 biologically independent experiments. Statistical significance was determined by one-way ANOVA with Tukey’s post hoc test (P < 0.05). (TIF) [file ppat.1013567.s004.tif]

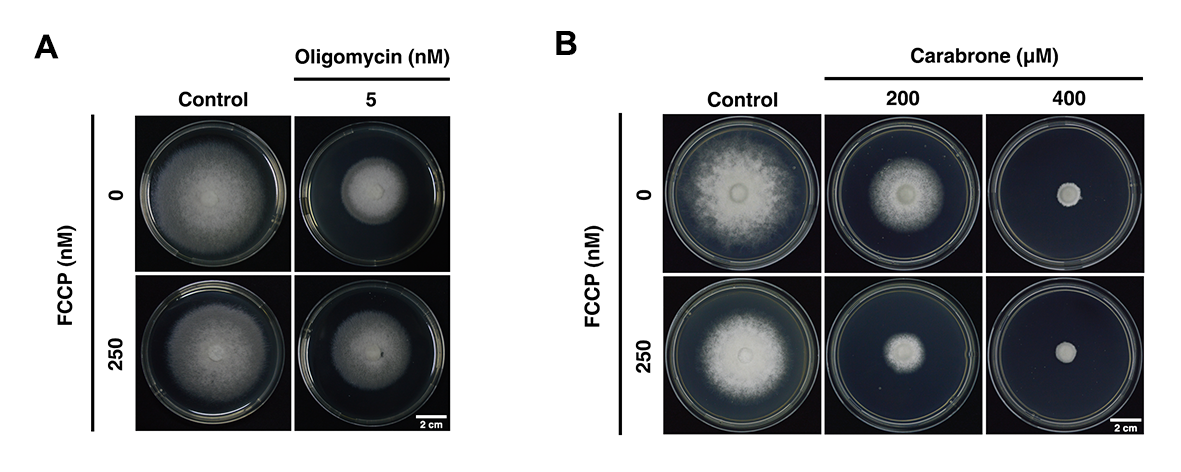

Supplement: S5 Fig — (TIF) [file ppat.1013567.s005.tif]

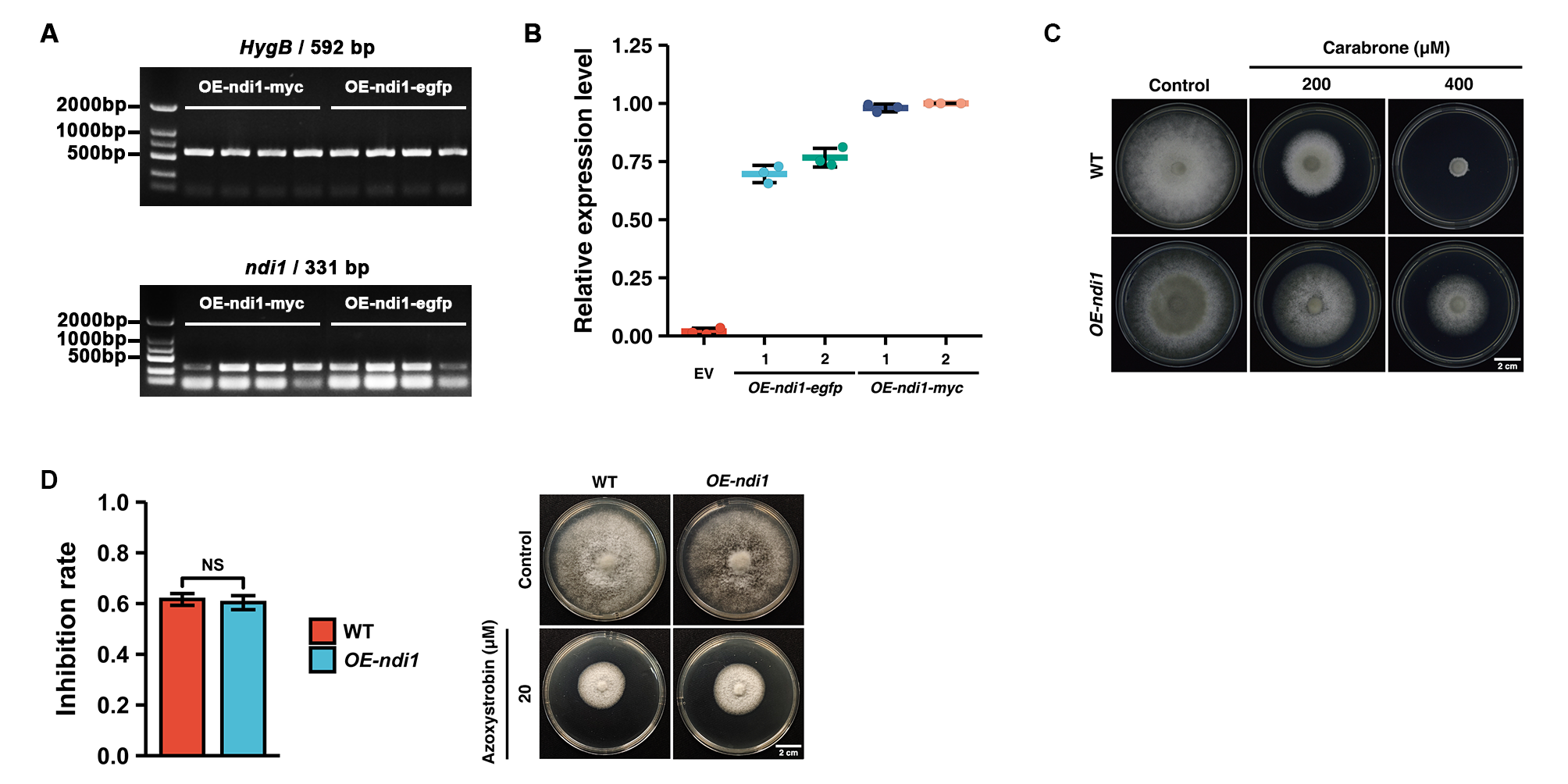

Supplement: S6 Fig — (A) PCR validation of the ndi1 overexpression strain in G. tritici. (B) RT-qPCR validation of the ndi1 overexpression strain in G. tritici. (C) Sensitivity of ndi1 overexpression strain to carabrone (200 and 400 μM). (D) Sensitivity of ndi1 overexpression strain to azoxystrobin (20 μM). Data are mean ± SD of n = 3 biologically independent experiments. Statistical significance was determined by one-way ANOVA with Tukey’s post hoc test (P < 0.05). (TIF) [file ppat.1013567.s006.tif]
