# Supplementary material for: Carabrone inhibits Gaeumannomyces tritici growth by targeting mitochondrial complex I and destabilizing NAD⁺/NADH homeostasis
Source: PLoS Pathog. 2025 Oct 3;21(10):e1013567. doi: 10.1371/journal.ppat.1013567 (PMC12510646; doi:10.1371/journal.ppat.1013567)
Supplement: S1 Text — (DOCX) [file ppat.1013567.s011.docx]

**Synthesis of the carabrone alkynyl probe (CAR-Y)**

To a round-bottom flask containing absolute ethanol (40 mL), carboxymethoxyamine hemihydrochloride (523.2 mg, 1.2 equiv), anhydrous sodium acetate (393.84 mg, 1.2 equiv), and carabrone (992.0 mg, 1.0 equiv) were added sequentially under a nitrogen atmosphere. The reaction mixture was stirred at room temperature for 12 hours. Upon completion, the reaction was filtered to remove precipitated solids. The filtrate was concentrated under reduced pressure and purified by silica gel column chromatography using a mixed eluent of petroleum ether/ethyl acetate/acetic acid (2:1:0.05) to afford **compound (1)**, which was used directly in the subsequent reaction.

**Compound (1)** was dissolved in dichloromethane (10 mL) and cooled to 0 °C in an ice bath. Separately, oxalyl chloride (dissolved in 1 mL dichloromethane) was slowly added dropwise to the stirred solution at 0 °C. After the addition, the reaction mixture was allowed to warm to room temperature and stirred for 3 hours. The solvent was then removed under reduced pressure to yield the corresponding acyl chloride intermediate. Acyl chloride was redissolved in dichloromethane (10 mL) and again cooled on ice. A solution of propargylamine and triethylamine (TEA) in dichloromethane (1 mL) was added dropwise to the cooled solution at 0 °C. The reaction mixture was stirred at room temperature for 3 hours. The reaction was quenched with 1 M hydrochloric acid (100 mL), and the aqueous layer was extracted with dichloromethane (3 × 10 mL). The combined organic layers were dried over anhydrous sodium sulfate, filtered, and concentrated under reduced pressure. The residue was further purified by silica gel column chromatography to afford the alkynyl-functionalized carabrone probe, named **CAR-Y**.

**Compound (1)**

Yield, 75%; Colorless oil; ^1^H NMR (500 MHz, DMSO-*d*_6_) δ 12.13 (s, 1H), 6.07 (d, *J* = 2.9 Hz, 1H), 5.72 – 5.68 (m, 1H), 4.90 – 4.81 (m, 1H), 4.47 (d, *J* = 7.8 Hz, 2H), 3.20 (s, 3H), 2.39 – 2.32 (m, 2H), 2.20 (q, *J* = 7.1, 6.6 Hz, 2H), 1.84 (d, *J* = 13.7 Hz, 4H), 1.56 – 1.47 (m, 1H), 1.48 – 1.37 (m, 1H), 1.07 (d, *J* = 7.0 Hz, 3H), 0.93 – 0.84 (m, 1H); ^13^C NMR (126 MHz, DMSO) δ 172.46, 171.80, 170.35, 158.54, 139.94, 122.59, 75.81, 75.78, 70.14, 49.07, 40.51, 40.43, 40.34, 40.26, 40.18, 40.10, 40.01, 39.84, 39.67, 39.51, 37.53, 37.07, 35.43, 33.58, 30.42, 30.39, 25.92, 22.87, 21.50, 18.41, 17.20, 14.70. MS-ESI *m/z* calcd for C17H23NO5[M+H]^+^: 321.16; found: 321.1637.

**CAR-Y**

Yield, 86%; Brown oil; ^1^H NMR (400 MHz, DMSO-*d*_6_) δ 8.04 (t, *J* = 5.7 Hz, 1H), 6.05 (d, *J* = 2.9 Hz, 1H), 5.68 (t, *J* = 2.6 Hz, 1H), 4.83 (ddd, *J* = 11.4, 8.8, 6.1 Hz, 1H), 4.35 (d, *J* = 8.5 Hz, 2H), 3.87 (td, *J* = 6.7, 5.8, 2.5 Hz, 2H), 3.17 (ddt, *J* = 9.3, 6.4, 3.3 Hz, 1H), 3.07 (dt, *J* = 12.1, 2.5 Hz, 1H), 2.44 – 2.25 (m, 2H), 2.16 (dt, *J* = 11.4, 6.7 Hz, 3H), 1.86 (s, 3H), 1.79 (s, 1H), 1.51 – 1.38 (m, 2H), 1.04 (d, *J* = 7.9 Hz, 3H), 1.00 – 0.81 (m, 2H), 0.54 – 0.45 (m, 1H), 0.35 (ddd, *J* = 8.7, 7.0, 4.1 Hz, 1H); ^13^C NMR (101 MHz, DMSO) δ 170.35, 170.33, 169.40, 159.25, 139.94, 122.64, 122.62, 81.68, 81.65, 75.79, 75.76, 73.24, 73.10, 72.33, 72.20, 40.90, 40.62, 40.41, 40.20, 39.99, 39.78, 39.57, 39.36, 37.51, 37.47, 37.05, 35.53, 33.57, 30.40, 28.09, 28.08, 25.91, 22.77, 20.08, 18.43, 17.17, 14.88. MS-ESI *m/z* calcd for C20H26N2O4[M+H]^+^: 358.19; found: 358.1910.
